# Digital alarm system with safety sensor and camera in special housing facilities—supportive or intrusive? A qualitative study of older adults’ experiences

**DOI:** 10.1186/s12877-026-07776-1

**Published:** 2026-06-13

**Authors:** Sanela Huskic Beslic, Catharina Gillsjö, Annelie J. Sundler, Mikaela Ridelberg, Jenny Hallgren

**Affiliations:** 1 School of Health Sciences, University of Skövde, Högskolevägen. Box 408, Skövde, 54128 Sweden; 2 College of Nursing, University of Rhode Island, Kingston, RI USA; 3 Faculty of Caring Science, Work Life and Social Welfare, University of Borås, Borås, Sweden; 4 The Swedish Red Cross University, Stockholm, Sweden

**Keywords:** Welfare technology, Digital alarm system, Safety sensor, Camera surveillance, Special housing facilities, Nursing homes, Older adults' experiences, Technological tolerance, Nursing care

## Abstract

**Background:**

The aging population is increasing due to advancements in medical research and public health, leading to a growing demand for healthcare services and special housing facilities (SHF). Welfare technology (WT) has been introduced to support older adults by enhancing safety, independence, and care efficiency. Despite its potential, WT adoption remains limited, and research on older adults' experiences, particularly in SHFs, is scarce. This study explores the experiences of older adults using a digital alarm system with a safety sensor and camera function (DASSCF) in SHFs.

**Methods:**

An inductive qualitative study was conducted using semi-structured interviews with older adults (3 men, 14 women, aged 67–98) in SHF equipped with DASSCF in a municipality in western Sweden. Data were collected between April and September 2024 and analyzed using qualitative content analysis.

**Results:**

Experiences of DASSCF resulted in *balancing vulnerability, tolerance, and having peace of mind in daily life* categorized into: emotional aspects, where DASSCF contributed to security in everyday life but also raised concerns about privacy; integrity aspects, highlighting the process of learning to live with surveillance and the tension between safety and autonomy; and technological aspects, which focused on usability challenges and the desire to have control over the system.

**Conclusions:**

When integrating DASSCF into SHF, WT should be as supportive as possible without being too intrusive, allowing older adults to feel strengthened while ensuring their safety and independence.

**Supplementary Information:**

The online version contains supplementary material available at 10.1186/s12877-026-07776-1.

## Introduction

The growing aging population is a result of advancements in medical research and public health [20]. Promoting the health and wellbeing of older adults, defined by the World Health Organization (WHO) as persons 65 years and above, is therefore essential and requires the provision of appropriate care alongside an enabling environment [37]. Aging leads to a gradual decline in biological functions, with increasing risk of functional impairments, frailty, hospitalizations and mortality [12]. It also affects mental and social wellbeing, heightening the risk of depression and social isolation [6]. Aging often involves multiple long-term health conditions and requires extensive health and social care. The ‘fourth age’ refers to the final stage of life, characterized by significant physical, and, to varying extents, cognitive decline. During this period, older adults’ experience increased frailty, diminished resilience and growing vulnerability to illness and disease, leading to a greater reliance on medical and social support [8]. As the aging population grows, the demand for healthcare services is rising [10], adding strains to an already overburdened healthcare system. Consequently, older adults may require extensive health and social care, some necessitating special housing facilities (SHF) for older adults. In Sweden, more than 80,000 older adults currently reside in SHFs [24] designed to accommodate older adults with long-term health conditions and complex healthcare needs [34]. Eligibility is determined through a formal needs assessment by the municipality, in accordance with the Social Services Act [33]. In the Swedish context, SHFs represent a form of residential care intended for older adults whose care needs have become too extensive to be managed in their ordinary homes. The resident population is heterogeneous, encompassing individuals with a range of conditions including cardiovascular disease, neurological disorders, dementia, and other forms of cognitive impairment, as well as significant physical frailty [22, 32]. Each resident occupies a private apartment, though shared spaces for meals and social activities are available within the facility [22]. Care is provided around the clock, by teams of registered nurses and care personnel, who together manage both social and healthcare needs [24, 32]. Occupational therapists and physiotherapists are available during daytime hours, and primary care physicians are consulted as needed. Facilities are operated either by the municipality directly or by private providers under municipal contracts, and residents contribute a regulated, income-based fee toward the cost of their care [8]. The procurement and financing of welfare technology (WT), such as DASSCF, falls under municipal responsibility and is generally funded as part of the broader care infrastructure [22, 32]. These SHF must balance the provision of care with upholding the older adult’s rights to privacy and autonomy, as they also serve as private homes [26].

Municipalities responsible for managing SHFs face significant challenges recruiting sufficient health and social care personnel to meet the growing demand for health and social care. The workforce shortage, particularly within social work and healthcare, places considerable pressure on the municipality, making it increasingly difficult to maintain quality care for all older adults [11]. To address the needs of older adults, innovative housing solutions, such as SHF equipped with integrated WT, have been developed [16]. In this context, WT can be an important contributor to social and healthcare provision [10, 17].

WT is defined as digital and technology-based interventions aimed at maintaining or improving safety, activity, participation, or independence for individuals who have, or are at increased risk of developing, a disability. These technologies also aim to increase efficiency in the delivery of social and healthcare services, while contributing to improved working conditions for social and healthcare personnel. Importantly, such interventions should be evidence-based [2, 25]. Stakeholders view WT as a significant potential contributor to the work of care personnel in caring for older adults; for instance, through the use of cameras for nighttime monitoring [28]. However, there are concerns among social and health care personnel regarding the use of cameras, such as the loss of social interactions [36], invasion of privacy, the potential misuse of surveillance, and the impact on the older adults’ dignity. These concerns highlight the ethical and practical dilemmas that arise when cameras are implemented in care settings [3]. Knowledge is limited regarding how care recipients perceive the social effects of WT when WT replaces health and social care personnel in SHF [4].

Despite their potential to address the challenges of caring for an aging population, many technological solutions in healthcare environments often fail to meet expectations when implemented in real-world settings [10]. Such technology enhances the safety of older adults, but is also perceived as intrusive and difficult to use. Although it is designed to promote independence, it sometimes has the opposite effect [16]. This discrepancy is partly due to a limited understanding of the complex relationship between technologies, older adults, and the context in which these solutions are applied [17].

Previous studies indicate that active involvement of older adults in determining the use [7, 18], development, and extent of WT contributes to an enhanced sense of wellbeing [1]. Encouraging a positive attitude regarding WT among older adults not only increases the likelihood of successful implementation but also enhances their willingness to adopt these technologies [39]. However, older adults often face difficulties in tolerating and utilizing new WT, due to several aspects such as lack of technical knowledge, fear of making mistakes, and concerns about reliability or complexity of the WT [11, 30]. Older adults' experiences show that these challenges are often linked to the technology's perceived intrusiveness and complexity, which can diminish their sense of autonomy and increase feelings of anxiety regarding the use of such systems [35].

A failure to adequately adapt WT to the specific needs and expectations of older adults has been shown to result in a gradual decline of older adults’ usage over time [4, 39]. When WT is poorly adapted older adults may disengage from the WT, which can in turn have negative consequences for their health and wellbeing. These findings underscore the importance of designing and implementing WT that is better tailored to the needs of older adults to effectively support their health and well-being, aligning with the principles of Person-Centered Care (PCC), where interventions are adapted to the individual. At the same time, despite the potential of WT to improve quality of life and care efficiency for older adults, its adoption remains limited [31]. WTs like safety sensors with surveillance cameras are commonly used to ease the burden on the healthcare system [27], yet there is limited empirical research on older adults' experiences with these technologies, especially in SHFs.

Moreover, older adults are not a homogeneous group, and standardized technological solutions often fail to meet individual needs [16]. While much research focuses on active older adults, there is a gap in understanding how WT impacts those in the fourth age, who often have complex care needs. This study aimed to explore the experiences of older adults using a digital alarm system with a safety sensor and camera function (DASSCF) in SHFs.

## Method

### Study design

An inductive qualitative study design was applied, based on semi-structured interviews with older adults in SHFs.

### Setting and participants

**Fig. 1:**
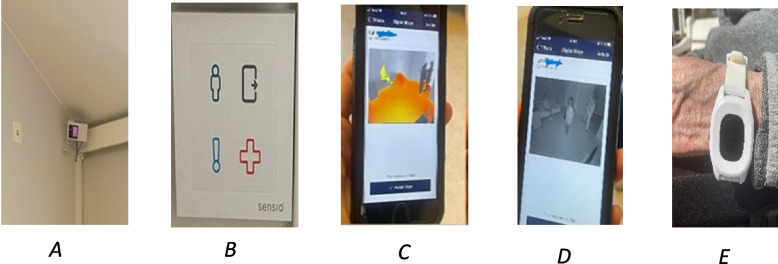
Photo collage of DASSCF

The study was conducted in SHFs for older adults equipped with DASSCF, in a municipality in western Sweden. Figure 1 provides an overview of the different components of the DASSCF, illustrating its structure and function within the SHF. The DASSCF system consists of a ceiling-mounted sensor equipped with a camera (A). A wall-mounted control unit (B) at the front door allows personnel to desable the alarm, register their presence with the patient (top left), leave the room (top right), request assistance from colleagues (bottom left) and trigger an emergency alert to colleagues (bottom right). Personnel members are equipped with mobile phones that display the identity of the person triggering the alarm. These devices also provide visual access to the residents’ rooms via the camera, with images appearing either blurred (C) or clear (D) depending on the residents’ chosen privacy settings. Residents carry an alarm button (E), worn either on the wrist or around the neck, which they can press to request assistance from personnel.

Unit managers in seven SHFs for older adults were invited by the head of health and social care to facilitate the recruitment of residents as study participants. Three units provided consent for participant recruitment, and a contact person was appointed by the unit manager at one of the three SHFs. The contact person was employed as a nurse assistant at one of the SHFs at the time of the study and had previous work experience from two of the participating units. The contact person was responsible for identifying potential participants in collaboration with the unit managers and care personnel at each SHF, based on the study’s inclusion criteria. Care personnel were not participants in the study but assisted in facilitating access to residents and supported the practical arrangements for the interviews. Inclusion criteria for study participation required that the older adults had been using the DASSCF for a minimum of six months and were able to participate in an interview conducted in Swedish. Each SHF housed approximately 30 older adults, all of whom utilized the DASSCF. When moving into the SHF, the older adults were asked to sign a consent form, authorizing the installation of the DASSCF and specifying the functionalities they desired, such as a fall alarm, activity detection such as rising out of bed, or being observed through the camera function in the safety sensor. A total of 3 men and 14 women agreed to participate in the study. All participants had some form of physical health condition that prevented them from continuing to live in their former ordinary housing, necessitating their residency in the SHF. These conditions included, but were not limited to, Parkinson’s disease, neurological disorders, mild cognitive impairment, degenerative diseases, and sequelae following stroke. All older adults lived by themselves in their own apartment at the SHF, and their ages ranged from 67–98 years.

### Data collection and procedure

Data were collected between April and September 2024. First, a digital meeting was held with the contact person and two of the researchers in order to share information about the study and procedures for data collection. Following this, the contact person communicated with the unit managers and social and healthcare personnel at each SHF for the inclusion of older adults according to the inclusion criteria. Older adults who met the inclusion criteria were first informed about the study verbally by the contact person, who asked if they were willing to participate. Interview appointments were then scheduled with those who consented. Prior to each interview, the first author provided both oral and written information about the study and obtained written informed consent from each participant. The first author visited each SHF at the agreed-upon time, and the interviews were conducted in the older adults’ apartments.

The interviews started with open questions concerning the older adults’ experiences of the DASSCF and the assistance they received from social and healthcare personnel. An interview guide (supplementary) was used, featuring open-ended questions such as: “Can you describe your experience with the new DASSCF in your residence?” and “How does the DASSCF affect your situation?” The responses were followed by probing questions to elicit deeper insights into individual experiences, such as: “Can you elaborate?” Each interview lasted approximately 15–40 min, depending on the participants’ condition and their ability to engage in the conversation. The interviews were audio recorded. All authors are registered nurses and researchers in the field of nursing science, with a specific focus on care of older adults, and have extensive experience in conducting qualitative interviews with this population.

### Analysis

The audio-recorded interviews were transcribed verbatim and analyzed using qualitative content analysis according to Graneheim and Lundman [15]. This method was chosen to develop a deeper understanding of older adults’ experiences of DASSCF, as well as how the DASSCF affects their sense of security and privacy. By using this approach, we aimed to identify key aspects and patterns that highlight the unique perspectives and emotions of the older adults in relation to DASSCF. The analysis aimed to shed light on the nuanced experiences of using DASSCFs, providing a contextualized and in-depth understanding of the phenomenon under study [15, 19]. The transcribed text was thoroughly read multiple times by the first author to understand the overall content. Meaning units related to the aim of the study were highlighted and extracted from the data. These units were abstracted and condensed to summarize their content and were subsequently assigned codes. The codes were then color-coded based on their association, differences, and similarities and organized into content areas, which formed the basis for developing subcategories and categories at a manifest level. An example of a sequence in the analysis is presented in Table 1. Three categories gradually emerged, and the codes were subsequently organized under each respective category. The categories were sorted into subcategories based on similarities and differences as described by Graneheim and Lundman [15]. A latent theme emerged representing the underlying message and shared meaning within the categories (Table 2). The quotes included in this article have been translated by the first author. The first author conducted the main analysis, but the process involved an iterative collaboration with co-authors, which further strengthened the credibility of the findings.

**Table 1:** Example of analysis steps

Meaning unit	Condensation	Code	Sub-category	Category	Theme
"Then I thought they wouldn’t come here because they would be seen on the camera. After that, I was able to fall asleep peacefully."	Thinking they’d be seen on camera; I could fall asleep peacefully	Feeling safe thanks to camera surveillance	Safety in everyday life	Emotional aspects	Balancing vulnerability, tolerance and having peace of mind in daily life

**Table 2:** Presentation of results

**Theme**	
Balancing vulnerability, tolerance and having peace of mind in daily life	

**Category**	**Subcategory**
Emotional aspects of life with DASSCF	Increased safety in case of fall incidents
	Comfort and security in everyday life
Integrity aspects of life with DASSCF	Learning to live with visibility
	Limiting one’s privacy
Technological aspects of life with DASSCF	Challenges in managing devices
	The desire to be in control of surveillance

### Ethical considerations

Ethical approval was obtained from the Swedish Ethical Review Authority in Gothenburg (Dnr. 2023-02063-01). The study was conducted in accordance with the Declaration of Helsinki [38] and the guidelines for nursing research in Nordic countries according to Northern Nurses’ Federation [29], as they serve as central ethical guidelines for medical research. The participants received information about the study, both orally and in writing, along with details on the procedures for participation and informed consent was obtained from all participants. The participants were also informed that they could withdraw from the study at any time with200out any consequences and without needing to provide an explanation.

## Results

The analysis resulted in the overarching theme “*balancing vulnerability, tolerance, and having peace of mind in daily life”* and included three categories: “emotional aspects of life with DASSCF”, “integrity aspects of life with DASSCF” and “technological aspects of life with DASSCF”.

The theme**,** *balancing vulnerability, tolerance, and having peace of mind in daily life,* was described as a complex challenge for older adults in SHF equipped with DASSCF. While the WT could contribute to a sense of safety by enabling a quick response from personnel when needed, the continuous monitoring could also be perceived as an intrusion into their privacy. Handling the DASSCF system was sometimes difficult for the older adult. It became a process of tolerating the benefits of WT as a way of maintaining safety and peace, while still wishing to retain their dignity and autonomy.

### Emotional aspects of life witH DASSCF

*Emotional aspects* address how DASSCF can enhance feelings of safety and security in SHFs. The subcategory *increased safety in case of incidents* highlights participants’ feelings in the event of falls or medical emergencies, when they want prompt support. The subcategory c*omfort and security in everyday life* emphasizes how surveillance can create a sense of security by keeping social and healthcare personnel aware of the participants' well-being, while also maintaining the respect and privacy desired by the participants.

#### Increased safety in case of fall incidents

The participants described that they have a need to feel safe in their daily lives, regardless of their physical condition. A common source of a lack of safety for the participants is the fear of falling and being unable to receive timely assistance, which negatively impacts their quality of life. The DASSCF was identified, by the participants, as a key element in enhancing their sense of safety offering the assurance of immediate assistance and continuous surveillance. One participant expressed the importance of this feature, stating:*”It is important for me to have the safety function, if I fall, they can see it and quickly come and help me”* (14). The sense of safety was further reinforced by the knowledge that personnel could be alerted and respond quickly, not only for the individual participant but for others in the facility as well. Another participant stated: *“There are many (older adults at the SHF) who have fallen, and received help quite quickly thanks to it (DASSCF)”* (4).

According to the participants, the DASSCF not only notifies care providers of incidents but also allows for automatic assistance if the participant is unable to activate the alarm manually, especially in cases where they are unable to rise after a fall. For some participants this was particularly significant given their experience of living alone in their apartments without immediate access to care personnel. This automatic function was described as particularly important, as some participants expressed concerns about situations in which they might lose the physical ability to call for help themselves. Knowing that the system could activate without their intervention provided an additional layer of reassurance, contributing to a sense of security even in the most vulnerable situations. This sense of security extended to everyday situations where participants were aware of their own risk-taking. One participant described how the awareness of the system provided reassurance even when consciously choosing not to use the walking aid: *"Sometimes I take a chance and do not bring my walker when I go to the bathroom, and then I feel that I might fall… but I know that if I fall, they (care personnel) will come"* (10). For these participants, the DASSCF thus provided not only reactive safety in emergencies but also a psychological safety net in the small risks of daily life.

#### Comfort and security in everyday life

The participants experienced an increased sense of security through the use of DASSCF, which not only ensures physical security by enabling monitoring and prompt assistance, when necessary, but also provides psychological reassurance. Some of the participants expressed that the DASSCF provided a sense of security in continuously tracking their overall condition, as they knew that health and social care personnel were able to observe them. One participant stated that the knowledge that someone can observe what is happening provides a sense of security: *“It is reassuring to live here when they can see me”* (5). The participants highlighted the comfort of knowing that help could be summoned without requiring manual activation, particularly in situations in which they might be incapacitated. Some of the participants did not currently make use of the DASSCF, but found peace of mind in its presence, anticipating its potential value in future scenarios such as cognitive impairment or emergencies. The participants also reflected on how the DASSCF contributed to a sense of security beyond physical incidents, particularly in relation to concerns about external threats. For some, thoughts about the camera’s protective function arose only in specific situations, and when they did, they were experienced positively. One participant described how watching frightening scenes involving violence or danger on television, had prompted reassuring thoughts about the camera: *“Then I thought they (potential intruders) wouldn’t come here because they’d be seen on the camera. After that, I could fall asleep peacefully”* (7). Others expressed that simply knowing the DASSCF was present, even without actively thinking about it, contributed to a general sense of calm and security in their daily lives.

### Integrity aspects of life with DASSCF

*Integrity aspects* explore perceptions of surveillance within SHFs equipped with DASSCF. The subcategory *learning to live with visibility* describes how the participants learn not to be consciously aware of the DASSCF in their daily lives. *Limiting one's privacy* highlights the potential sense of intrusion into personal space that surveillance might bring.

#### Learning to live with visibility

Participants described initial feelings of discomfort, when first moving in and starting to use the DASSCF, but these sensations diminished over time as they adapted to its presence. A few participants described the process of learning how to deactivate the sensor when needed, such as during visits, as an important part of this adaptation, as it allowed them to maintain privacy and prevent unnecessary monitoring during certain times: *"…but they’ve taught me how to turn it off when the kids (grandchildren) are here… they jump and bounce around. The care personnel came in several times in a hurry… but now I’ve learned to turn it off when I have visitors…”* (3) Other participants described how they gradually tolerated the presence of the DASSCF, by reflecting that care personnel likely have more important tasks than constant surveillance which helped alleviate concerns about intrusion. As a result, they expressed little concern about the DASSCF as an intrusion on their integrity, indicating, that over time, they became more comfortable with the system and its role in their lives.

Over time, the sensor was described by most participants as discreet and unobtrusive, rarely noticed in daily life. Its placement high on the ceiling and its silent operation further reinforced this sense of invisibility. While some participants expressed concerns about the DASSCF’s capacity to monitor their activities, the majority reported feeling neutral or indifferent, acknowledging an awareness of the sensor but considering it insignificant in their daily routines. Some participants drew a distinction between passive awareness and active discomfort, knowing the sensor was there without feeling watched, which allowed them to engage in their usual activities without a sense of being constantly under surveillance. For these participants, the DASSCF had been integrated into daily life in a way that minimized anxiety rather than amplifying it, a process supported by the possibility to tailor its use to their individual needs and circumstances. One participant captured this sentiment:

“I do not think much about the sensor, to be honest… it’s just there, and it’s not something I dwell on or think about … they can see me, and that’s fine…” (3)

This reflects how the process of adaptation, for some participants, resulted in the DASSCF becoming a natural and largely unnoticed part of their everyday living environment.

#### Limiting one’s privacy

The tolerance of camera surveillance among the participants was context-dependent. The participants indicated that they would not consent to such surveillance if they were living in their former homes. Their tolerance of surveillance within the SHF was associated with their specific living conditions and altered health status, rather than with a general willingness to tolerate surveillance. The transition to the SHF prompted by declining mobility, influenced their perception of the DASSCF.

While some participants expressed concerns about camera surveillance in DASSCF, viewing it as an infringement on their privacy, others articulated strong dissatisfaction with continuous monitoring. They perceived this constant observation as a restriction on their personal privacy and autonomy. This sentiment was particularly prevalent among those who felt that, despite residing in the facility voluntarily and paying for their accommodations, they did not agree with the notion of being perpetually monitored.

"I think it’s an invasion of privacy. I live here voluntarily… I pay rent… but I don’t want to pay for an alarm that constantly monitors what I do." (14)

Some participants described how the experience of surveillance extended beyond the camera itself, reflecting a broader sense of lost privacy in daily life. One participant expressed that the combination of camera monitoring and personnel entering without waiting contributed to an overall feeling of having no freedom: *"I feel monitored… no freedom… and you don't really have that anyway, because they knock and then just walk right in."* (15).

Some participants described experiencing a sense of captivity, reporting that the presence of DASSCF made the residence feel less like a personal home and more like a confined space with restricted freedom. The familiar and cozy qualities that make a place feel like home were described as diminished by the constant presence of monitoring, with some participants noting that the cameras contributed to a more institutional atmosphere. One participant even characterized the environment as resembling a prison, where constant monitoring creates a feeling of being under continuous control and observation: *"If I lived at home, I wouldn’t have a camera like that… the only reason I came here was that my mobility got worse… and now I’m in a wheelchair…"* (5). Another participant remarked that the surveillance evoked memories of a hospital environment, contributing to an unpleasant and unsettling atmosphere. Reactions to the surveillance varied among the participants; where some expressed little concern about the cameras, others reported a heightened awareness of being monitored, reflecting the highly individual nature of how privacy was experienced within the facility.

### Technological aspects of life with DASSCF

*Technological aspects of life with DASSCF* described the challenges that the participants in SHFs face when interacting with DASSCF. The subcategory c*hallenges in m*anaging *devices* addresses various issues that may arise, including system malfunctions and complex interfaces that hinder effective use. This uncertainty experienced by the residents in using the DASSCF can diminish confidence in the system, ultimately affecting their overall sense of safety and security within the care environment. *The desire to be in control of surveillance* addresses the participants’ need to understand and exert some control over when and how monitoring occurs, fostering a sense of involvement and autonomy.

#### Challenges in managing devices

The participants in the SHF described various challenges related to the use of alarm buttons, which form a part of the DASSCF, primarily due to a combination of limited knowledge of the system and physical impairments. A common issue was a lack of understanding of the DASSCF's functionality, leading to uncertainty about whether their alerts had been successfully sent, which sometimes resulted in delays receiving assistance during emergencies. Physical impairments, such as reduced mobility, coordination difficulties, arthritis and muscle weakness, also made it difficult to reach and press the alarm button effectively as the physical design did not accommodate diverse needs of the participants, requiring repeated or forceful pressing to activate: *"Yes, they came eventually, but I had to keep trying and pressing for quite a while before I succeeded*." (2). These challenges led to anxiety about whether they would be able to activate the alarm in an emergency, particularly in situations where quick assistance was required.

Several participants described the alarm button in DASSCF as overly sensitive, resulting in frequent accidental activations and misunderstandings with care personnel. The lack of clear feedback made it difficult to verify whether the button had been pressed correctly, contributing to feelings of confusion and insecurity: *"It’s (the alarm button) so sensitive… and it’s difficult with my hands… and it’s easy to press accidentally… so it could go off on its own… but they (care personnel) are aware of that by now." (3).* Beyond sensitivity issues, some participants also described situations where the alarm failed to register entirely, leaving them without assistance when needed. This created a profound sense of vulnerability, as one participant described: *"If I want help and the regular alarm does not work… and I need help, something has happened to me… and there is no one responding to it."* (13). Some participants noted that they occasionally activate the alarm button accidentally, which sometimes occurred without their awareness. One participant described it as follows:

"It doesn’t feel good that they just come in and say that I’ve set off the alarm… and I’m not aware that I have. No, I haven’t set off the alarm. Yes, you have; we can see it here (the care personnel says)… But I don’t see it, and they say I’ve set off the alarm… it’s wrong." (1)

The participants suggested several improvements, including ergonomic adjustments, better feedback mechanisms, clearer instructions, and a simplified usage process, emphasizing that a design better adapted to physical and cognitive limitations would improve both reliability in emergencies and their overall sense of autonomy and wellbeing.

#### The desire to be in control of the surveillance

The participants had mixed feelings about the visibility and functionality of the system. While some found it reassuring, others expressed uncertainty about how the surveillance operates and about when the camera is active. This lack of clarity led to concerns about privacy and security, particularly regarding the visibility of the system’s signals. Several participants mentioned that not knowing when they were being monitored led to discomfort and a sense of unease. A clear indicator of when the camera is active was seen as an essential feature for improving trust in the system. Participants felt that a visible signal, such as a light, could help distinguish when the monitoring was occurring, reducing feelings of uncertainty and enhancing their sense of control. *"Yes… I wonder if it could light up green, perhaps, when they’re looking… I’m not sure… It should be able to do that at night, at least, so I can see it glowing." (3).*

Beyond visual indicators, the ability to control when the camera is on or off was also highlighted as a key factor in fostering a greater sense of security. The participants speculated that having control over activation could reduce potential anxiety, as it would allow them to manage their own privacy. This, in turn, could contribute to a more comfortable and autonomous living environment. *"Yes, when they look at me, I would like it to light up in a different color… so I can see that they are looking at me now."* (1).

Additionally, some participants indicated a lack of understanding about the overall functionality of the system, particularly regarding how and when surveillance is conducted. They emphasized the need for clearer information on how the system operates, as greater transparency would likely improve trust and confidence in its use. Providing explicit guidance on the surveillance process and ensuring that visual indicators are intuitive were suggested as ways of enhancing the overall experience and sense of control.

## Discussion

The findings of this study show that living with DASSCF in SHF is a complex experience, shaped by competing needs for safety and privacy. Older adults’ perceptions of security, privacy, and autonomy are significantly impacted. These findings examine the multifaceted effects of DASSCF, exploring emotional responses like feelings of safety or discomfort, integrity concerns related to autonomy and dignity, and technological issues such as system usability and control. These interconnected dimensions influence how older adults experience and tolerate the presence of surveillance in their living environments. A key finding of this study is the considerable variation in how older adults perceive and adjust to DASSCF, underscoring the importance of tailoring care to each individual's needs and preferences. This highlights the need for a person-centered approach [21], where technology is implemented in a way that respects and supports individual differences and autonomy.

The findings illustrate how DASSCF can offer quick responses in case of emergencies for older adults needing assistance. Older adults’ need for assistance and security in daily life, may lead to ambiguous experiences, where vulnerability in old age can be related to an insecurity and fear of falling and to not being able to receive help in time when needed. DASSCF can be a valuable resource in managing such vulnerability, offering both immediate assistance and the possibility of continuous monitoring. These findings are consistent with previous research showing that technological solutions, particularly alarms and monitoring systems, can foster a sense of security for older adults [31, 35], who might otherwise fear being left alone in the event of an incident, such as in the case of a fall [35].

The findings also reveal how the older adults initially felt discomfort when using and living with the DASSCF, that it was perceived as an intrusion on their privacy. However, this discomfort decreased over time, indicating that participants adapted to the presence of the DASSCF. An interesting aspect is that some participants highlighted the importance of being able to deactivate the DASSCF when needed, such as during visits, suggesting that the ability to exercise autonomy and control is a key factor in reducing the feeling of being monitored. This highlights the significance of person-centered care, which prioritizes personal preferences, autonomy, and shared decision making [9]. This perspective also aligns with previous research, such as Boström et.al. and Tham et al. [5, 35] who emphasize the importance of giving older adults the opportunity to actively participate in decisions regarding the use of WT. Ensuring that these older adults have agency in these decisions not only respects their autonomy but also fosters a sense of dignity and trust in the care environment. By tailoring WT to personal needs and preferences, social and healthcare personnel can support a more person-centered approach, enhancing both tolerance of WT and overall wellbeing [1, 7, 9, 18]. Person-centred care (PCC) provides a valuable theoretical lens for understanding older adults' experiences of DASSCF. According to McCormack and McCance's Person-Centred Nursing Framework [21], care should be grounded in a therapeutic relationship that respects the person's values, beliefs, and individual needs. Within this framework, the implementation of WT such as DASSCF should not merely serve organizational efficiency, but actively support the older adult's sense of identity, autonomy, and dignity. The findings of this study reflect core tensions within PCC. While DASSCF enhanced feelings of safety and security, values expressed by participants, the experience of continuous surveillance simultaneously challenged participants' sense of autonomy and privacy, which are equally central to PCC. This tension underscores the importance of shared decision making, where older adults are actively involved in determining how and when surveillance technologies are used in their homes [7, 21]. For DASSCF to support PCC in practice, it would therefore require not only technically flexible systems, but also ongoing dialogue between older adults and care personnel to ensure that the technology remains aligned with individual preferences and life circumstances. The diverse reactions observed in this study further illustrate that a one-size-fits-all approach is not effective in practice. Taken together, these findings reinforce the value of PCC as a guiding framework when implementing DASSCF, one that calls for personalized, adaptable solutions that place the older adult’s voice and lived experience at the centre of care.

Furthermore, several older adults were found to perceive the sensor as neutral or insignificant in their daily lives over time. This may be because the DASSCFs' functions were viewed as supportive rather than as intrusive. Previous research also indicates that older adults often acclimate to welfare technologies due to a lack of alternatives to traditional solutions in health and social care. Such adaptation often stems from necessity rather than active choice, which may influence their perception of autonomy and independence [13]. Some participants also reflected on the likelihood that care personnel have more important tasks than continuously monitoring them, which helped alleviate concerns about intrusions into their privacy. This suggests that perceived trust in care personnel and their priorities plays a role in how the sensor is received. For instance, when implementing remote DASSCF, healthcare personnel must remain attentive to safety concerns such as system reliability and emergency response time as well as potential privacy violations, including unauthorized access. Failing to address these issues could undermine older adults’ trust and negatively impact their overall experience [40].

The results also highlight the nuanced perceptions of older adults regarding camera surveillance in SHFs, while some perceive such monitoring as a safety-enhancing arrangement, others see it as an infringement on privacy and autonomy. It has also been emphasized in previous studies that despite the intended safety benefits, surveillance cameras can evoke feelings of intrusion and discomfort among older adults [10]. The presence of surveillance technologies can alter the perception of 'home' for older adults, potentially impacting their sense of security and self-confidence. As home represents the place of central importance to older adults, where they feel most at ease and can preserve their identity, integrity and way of life [14], there is a critical need to prioritize older adults’ psychological wellbeing when implementing monitoring solutions [23]. This variation in perception of surveillance and privacy further underlines the need for a flexible, individualized approach to the implementation of WT, ensuring that the specific needs and preferences of each older adult are considered to enhance their well-being and autonomy.

Data collection for this study was conducted over a period of four months, which is considered adequate duration for data gathering. The same main questions were asked as the researcher followed an interview guide. Prolonged data collection periods may lead to inconsistencies, such as the introduction of new interview questions or the alteration of participant interactions [15]. To enhance dependability, efforts were made to ensure consistency in data collection by adhering to a structured interview guide while allowing for natural variations in responses. In studies on older adults and WT, there is a risk of sampling bias. The older adults who chose to participate in the interviews may be more positive or open to WT than those who chose not to participate, which can affect the results. This could lead to an overrepresentation of positive viewpoints, while those who are more skeptical or hesitant about WT may be underrepresented. It is therefore important to consider this potential bias, as the results may not fully reflect the opinions and experiences of the entire older population. The sample size in this study is relatively small, which limits the ability to draw general conclusions about the experiences of older adults. However, it is unlikely that the results would have differed with a larger sample, as the collected responses were nuanced and included both positive and negative experiences. The credibility of the findings was strengthened by the transparency of the analysis process and the use of quotations, which provided a deeper insight into participants’ perspectives [15]. Regarding transferability, the findings are specific to the particular WT used in the studied SHFs and therefore cannot be considered representative of WT in general. However, by providing a clear description of the study context, participants’ characteristics, and the data collection process, the study offers valuable insights that may be applicable to similar settings. A rich presentation of findings**,** supported by direct quotations, further enhances the potential for transferability.

## Conclusion

The integration of DASSCF into the lives of older adults presents both opportunities and challenges. While these technologies can enhance independence, improve perceived sleep quality, and increase a sense of safety, their implementation must be carefully managed to address concerns related to privacy, security, and autonomy. Older adults' experiences of DASSCF show that when these systems are introduced in a way that actively involves them to tailor the WT to their needs, it can minimize feelings of intrusion and allow them to maintain control over its use. WT should be as supportive as possible without being too intrusive, allowing older adults to feel strengthened while ensuring their safety. Similarly, the adoption of DASSCF in SHFs brings emotional, integrity-based, and technological challenges that shape how older adults experience and adapt to these systems. A respectful, individualized approach to implementation is essential to ensure that these technologies support, rather than undermine, the well-being and dignity of older adults.

## Supplementary Information

Supplementary Material 1.

## Data Availability

The datasets generated and analyzed during the current study are not publicly available due to ethical considerations and the terms approved by the Swedish Ethical Authority. The participants did not provide consent for their data to be shared beyond research team. However, data not comprising confidential information are available from the corresponding author on reasonable request.
