# Supplementary material for: Digital alarm system with safety sensor and camera in special housing facilities—supportive or intrusive? A qualitative study of older adults’ experiences
Source: BMC Geriatr. 2026 Jun 13;26:839. doi: 10.1186/s12877-026-07776-1 (PMC13267262; doi:10.1186/s12877-026-07776-1)
Supplement: Supplementary file 1 — Supplementary Material 1. [file 12877_2026_7776_MOESM1_ESM.docx]

**How do residents experience the new alarm system with a safety sensor in XX Municipality?**

**Demographic Data:**

- Age, Gender, Family, Work Life, Current Residence, Time in Residence

*Describe your experience with the new alarm system and the safety sensor in your residence.*

- What information did you receive about the alarm before its implementation? Was consent obtained?

*How does the new alarm system function?*

- What features are available? Which features are used? (alarm button?)
- What issues have arisen? How were they resolved?

*How does the safety sensor function?*

- What features are available? Which features are used? (fall alarm, bed exit alarm?)
- What issues have arisen? How were they resolved?

*How does the alarm system impact your situation?*

- Changes in the living environment
- Changes in personnel working methods
- Assistance from personnel – waiting time

*How does the safety sensor impact your situation?*

- Changes in the living environment
- Changes in personnel working methods
- Assistance from personnel – waiting time

*What impact does the alarm system have on your experience of the care you receive?*

- Health and well-being (e.g., sleep quality)
- Sense of security, independence

*What impact does the safety sensor have on your experience of the care you receive?*

- Health and well-being (e.g., sleep quality)
- Sense of security, independence

*Has the new alarm system with the safety sensor affected your relatives?*

- If yes, in what way?

*How do you view the need for further development of the alarm system and safety sensor?*
